# Comparison of perfluorocarbon liquid utilization in endoscopic pars plana vitrectomy and microscopic pars plana vitrectomy during retinal detachment repair: a retrospective review

**DOI:** 10.1186/s40942-026-00802-9

**Published:** 2026-02-12

**Authors:** Amanda K. Hertel, Sarah Frederick-Fisher, Joshua Fernandes, Maram El-Geneidy, Riya Parikh, Shreeya Dalla, Matthew M. Pfannenstiel, Anthony Meljanac, Smith Asoh, Madeleine Mueller, Radwan S. Ajlan

**Affiliations:** Department of Ophthalmology, The University of Kansas School of Medicine, Kansas City, KS USA

**Keywords:** Retinal detachment (RD), Pars plana vitrectomy (PPV), Endoscopy, Perfluorocarbon liquid (PFCL), PFO

## Abstract

**Purpose:**

Retinal detachment (RD) is a vision threatening condition that often requires surgical intervention. During RD repair, perfluorocarbon liquid (PFCL) use, such as perfluoro-n-octane (PFO), can be used as a retinal tamponade source. However, PFO use adds additional cost and may lead to complications like inflammatory response. Pars plana vitrectomy (PPV) can be done the standard method using a surgical-microscope PPV (S-PPV) or with endoscopic PPV (E-PPV) in cases of hazy anterior view. The purpose of this study is to compare PFO use and outcomes between E-PPV and S-PPV for RD repair.

**Methods:**

A retrospective chart review of patients undergoing RD repair between August 2017 and July 2023 at a tertiary referral center was performed. Patients were included if they had a minimum of 6-months postoperative visits. Data was collected for patient demographics, visual acuity (VA), intraocular pressure (IOP), procedure details, and surgical outcomes. Statistical analysis was performed using t-tests and chi-square analysis. This study was approved by our institutional review board (IRB).

**Results:**

401 patients met the inclusion criteria with an average age of 59.17 (SD = 14.66). Of those included, 38.15% were female and 61.85% were male. PFO was utilized in 29.18% (*N* = 117) of cases. Most procedures performed were S-PPV (80.05%, *N* = 321) with the remaining cases being E-PPV (19.95%, *N* = 80). PFO was utilized in 38.75% of E-PPV cases, but in only 26.79% of S-PPV cases (*p* = 0.035). There were some variations in VA and IOP outcomes, with the PFO group generally having worse preoperative visual acuity. There were no statistically significant differences in rates of retinal re-detachment, cystoid macular edema (CME), or epiretinal membrane (ERM) development between any of the groups.

**Conclusions:**

In this study, PFO was used more frequently in those undergoing E-PPV compared to S-PPV. With PFO use, there was no statistically significant difference in re-detachment rate between the E-PPV and S-PPV groups. PFO use may be more beneficial in patients with poor anterior view given its higher utilization rate in E-PPV.

**Supplementary Information:**

The online version contains supplementary material available at 10.1186/s40942-026-00802-9.

## Introduction

Retinal detachment (RD) is a sight-threatening condition most commonly repaired by pars plana vitrectomy (PPV) in the United States [1, 2]. In complex cases, surgeons frequently use intraoperative perfluoro-n-octane (PFO), a dense liquid that effectively stabilizes and repositions the retina [3, 4]. PFCl use can be planned pre-operatively like in the repair of giant retinal tears (GRT) or planned retinectomy for PVR. It may also be decided upon intraoperatively to assist in subretinal fluid drainage, or stabilizing the retina [3, 4]. Despite its surgical advantages, PFO is associated with potential toxicity, postoperative inflammation, and a significant increase in procedural cost, limiting its routine use [5–7].

The decision to employ PFO can be influenced by the surgical visualization technique itself. Vitrectomy is typically performed using a surgical-microscope PPV (S-PPV) but requires endoscopic PPV (E-PPV) in cases with anterior segment opacity [8]. Unlike the fixed microscope, E-PPV demands that the surgeon manually maintain stable visualization with the endoprobe. This inherent instability may necessitate a greater reliance on PFO to flatten and secure the retina, potentially leading to different utilization patterns between the two techniques.

This potential difference in surgical practice, however, remains unquantified in the literature. To date, no studies have specifically evaluated the rate of PFO use in E-PPV compared to S-PPV for retinal detachment repair. This study aims to address this knowledge gap by comparing PFO utilization rates between the two approaches. Furthermore, we will assess how PFO use impacts key outcomes, including final visual acuity (VA), intraocular pressure (IOP), and re-detachment rates.

## Methods

This retrospective cohort study was conducted at a single, tertiary referral center and received approval from the Institutional Review Board (IRB) under study ID [00143338]. The study included adult patients (≥ 18 years of age) who underwent surgical repair for retinal detachment (RD) between August 2017 and July 2023. To be included, patients were required to have a minimum of six months of postoperative follow-up data available.

Patient data were extracted from electronic medical records. Collected variables included patient demographics, best-corrected visual acuity (BCVA), intraocular pressure (IOP), and the primary exposure variable: intraoperative PFO use. The primary surgical outcome was the rate of retinal re-detachment. Secondary outcomes included the development of cystoid macular edema (CME) and epiretinal membrane (ERM) formation. For all E-PPV procedures, a straight 23-gauge endoscope with a 10,000-pixel image resolution was utilized.

For analysis, all BCVA measurements were converted to LogMAR values $\:LogMAR\:BCVA=-\mathrm{l}\mathrm{o}\mathrm{g}\left(decimal\:acuity\right)$ [9, 10]. Standardized LogMAR values of 2.6, 2.7, and 2.8 were assigned for counting fingers, hand motion, and light perception, respectively [11, 12]. Continuous variables, such as LogMAR VA and IOP, were compared between groups using two-sample t-tests. Categorical outcomes, including complication rates, were analyzed using the chi-squared test. A p-value of less than 0.05 was considered statistically significant for all analyses.

## Results

A total of 401 patients met the inclusion criteria. The cohort had a mean age of 59.0 years (SD = 14.7) and was predominantly male (61.9%). The majority of patients had rhegmatogenous detachments (63.5%, *N* = 255) and the remaining had tractional detachments (36.4%, *N* = 146). Some of the patients with rhegmatogenous detachments also had giant retinal tears (GRT) (8.7% of entire cohort, 13.7% of RRD cohort, *N* = 35). PFO was used in 69.8% (*N* = 178) of the RRD repairs and in the majority of cases with GRT (65.7%, *N* = 23). Of those with tractional detachments, 45.2% had PVR (*N* = 66). PFO was used in 28.8% (*N* = 42) of the TRD cases. Most were partial RD, with minority having total RD (2.0%, *N* = 8). Trauma caused 4.7% (*N* = 19) of the RDs in the study. Additional information such as number of tears or grade of PVR were not included due to the retrospective nature of this study. Most retinal detachment repairs were performed using standard PPV (S-PPV; 80.1%, *N* = 321), with the remainder performed using endoscopy-guided PPV (E-PPV; 20.0%, *N* = 80). E-PPV was used more frequently in those with TRD (29.5%, *N* = 43) compared to RRD (14.5%, *N* = 37). The overall rate of retinal re-detachment for the entire cohort was 7.98% (*N* = 32), with most events (81.3%) occurring within the first month postoperatively. Other complications were infrequent, with cystoid macular edema (CME) in 1.2% (*N* = 5) of patients and epiretinal membrane (ERM) formation in 1.0% (*N* = 4). Additionally, around a quarter (28.7%, *N* = 115) experienced proliferative vitreoretinopathy (PVR). Detailed demographics and results are in (Tables 1 and 2). Additional data can be found in (Supplementary Tables 1–5).

**Table 1:** Patient demographics and procedure performed (*N* = 401)

Total	*N* = 401
Gender	N (%)
Female	153 (38.15)
Male	248 (61.85)
Mean age in years (SD)	59.17 (14.66)
PFO Utilization	N (%)
PFO	117 (29.18)
No PFO	284 (70.82)
Endoscopy utilization	N (%)
Endoscopy	80 (19.95)
No Endoscopy	321 (80.05)
PFO + Endoscopy utilization	N (%)
PFO with Endoscopy	31 (7.73)
PFO without Endoscopy	86 (21.45)
No PFO with Endoscopy	49 (12.22)
No PFO without Endoscopy	235 (58.60)

**Table 2:** Rates of RD, CRM, and ERM after Postoperatively. N (%)

	RD	CME	ERM	PVR
All (*N* = 401)	32 (7.98)	5 (1.25)	4 (1.00)	115 (28.68)
PFO (*N* = 117)	12 (10.26)	2 (1.71)	2 (1.71)	48 (41.03)
No PFO (*N* = 284)	20 (7.04)	3 (1.06)	2 (0.70)	67 (23.59)
E-PPV with PFO (*N* = 31)	4 (12.90)	0 (0.00)	0 (0.00)	18 (58.06)
S-PPV with PFO (*N* = 86)	10 (11.63)	2 (2.33)	2 (2.33)	30 (34.88)
E-PPV without PFO (*N* = 49)	1 (2.04)	1 (2.04)	0 (0.00)	26 (53.06)
S-PPV without PFO (*N* = 235)	19 (8.09)	2 (0.85)	2 (0.85)	41 (17.45)

RD = Retinal Detachment, CME = Cystoid macular edema, ERM = Epiretinal membrane formation, PVR = Proliferative vitreoretinopathy

Overall, perfluoro-n-octane (PFO) was used in 29.2% (*N* = 117) of all surgeries. A key finding was the difference in utilization based on surgical technique: PFO was used in 38.8% of E-PPV procedures compared to 26.8% of S-PPV procedures (*p* = 0.035).

When comparing all patients who received PFO (*N* = 117) with those who did not (*N* = 284), the PFO group had significantly worse preoperative visual acuity (logMAR 1.71 vs. 1.31, *p* < 0.001). This difference in vision persisted at all postoperative time points from one week to one year. The No PFO group had a slightly higher preoperative IOP (*p* = 0.006), with no significant differences in IOP at any postoperative follow-up. There were also no statistically significant differences in the rates of retinal re-detachment (10.3% vs. 7.0%, *p* = 0.280), CME (1.7% vs. 1.1%, *p* = 0.592), or ERM formation (1.7% vs. 0.7%, *p* = 0.357). There was some difference in PVR between the two groups (41.0% vs. 23.6%, *p* = 0.00045).

Within the E-PPV sub-group, patients receiving PFO (*N* = 31) had significantly worse preoperative VA than those without PFO (*N* = 49) (logMAR 2.23 vs. 1.73, *p* = 0.025). There was a trend toward a higher re-detachment rate in the PFO group (12.9% vs. 2.0%), which was marginally significant (*p* = 0.051). There were no significant differences in other outcomes. Similarly, within the S-PPV sub-group, patients receiving PFO (*N* = 86) had significantly worse preoperative VA than those without PFO (*N* = 235) (logMAR 1.50 vs. 1.19, *p* = 0.026). There were no statistically significant differences in rates of retinal re-detachment (11.6% vs. 8.1%, *p* = 0.327) or other complications between the subgroups except for rates of PVR. PVR was higher in the S-PPV with PFO group (34.9% vs. 17.5%, *p* = 0.000857).

To compare the presumed most and least complex cases, the E-PPV with PFO group (*N* = 31) was compared to the S-PPV without PFO group (*N* = 235). The E-PPV with PFO group had worse preoperative VA (logMAR 2.23 vs. 1.19, *p* < 0.001). This significant difference in vision persisted through the one-year follow-up (*p* < 0.001). Despite the significant differences in baseline vision, there was no statistically significant difference in the rate of retinal re-detachment (12.9% vs. 8.1%, *p* = 0.370) or other complications between these two extreme groups except for a difference in PVR. The E-PPV with PFO group had higher rates of PVR (58.1% vs. 17.5%, *p* < 0.00001).

## Discussion

Our study revealed two principal findings: first, perfluoro-n-octane (PFO) was utilized significantly more often in endoscopy-guided PPV (E-PPV) than in standard microscopic PPV (S-PPV) (*p* = 0.035). Second, while patients receiving PFO consistently had worse baseline visual acuity, they ultimately achieved comparable rates of surgical success compared to the non-PFO group. When comparing the different sub-groups, there were no statistically significant differences between rates of retinal re-detachment, CME, or ERM.

PFO has a high specific gravity allowing it the reposition and stabilize the retina during vitreoretinal surgery [3]. Thus its use can improve control during surgery and also ultimately save time [3].

PFO use increases the average cost of a PPV by up to a 21% [6]. The use of PFO can also lead to complications such as retained PFO in 1–3.5% of cases [13]. Early studies have found that small amounts of retained PFO are generally well-tolerated and not retina toxic [14]. However, when retained for longer or in larger amounts, the presence of PFO leads to white precipitates throughout intraocular structures [14] and other negative effects such as elevated intraocular pressure [15] or delayed inflammatory response [16].

During E-PPV in particular, PFO is beneficial as it stabilizes the retina, displaces subfoveal blood, and allows for better endoscopic visualization during vitreous hemorrhage as it does not mix with it. This benefit must be weighed against the added cost, and potential toxicity of PFO. However, when compared to potential alternative solution of using a temporary keratoprosthesis and subsequent penetrating keratoplasty, the added PFO cost, and risks diminish. It is also noted that the cases requiring use of endoscopy and PFO were likely more complicated at baseline. Some may believe the outcome would be worse in the E-PPV group since they would be expected to have more complex and complicated detachments. However, there is currently limited literature to support this. In our study, we found comparable rates of surgical success between the E-PPV and S-PPV groups.

The rates of PVR were much higher in the groups receiving PFO. Studies have suggested that the use of PFO may be beneficial in complex RD repair complicated by PVR [17, 18]. The increased number of PVR in E-PPV cases may contribute to the increased use of PFO in E-PPV.

A strength of this study is the large sample size of over 400 patients undergoing PPV procedures for retinal detachment. Patients were also followed over multiple postoperative visits, allowing data to be collected on visual acuity, IOP, and any resulting complications. One limitation is the retrospective nature of this study. Additionally, all data was collected from one academic center because of limited number of centers performing similar cases locally. PPV procedures were performed by a single retina surgeon which may help minimize bias implied by multiple surgeon’s surgical results. Further studies are needed across multiple institutions to determine generalizability of this data.

## Conclusion

In conclusion, PFO is more frequently employed in E-PPV and appears to be a tool reserved for more complex retinal detachments. When utilized in E-PPV, it enabled these difficult cases to achieve a surgical success rate comparable to that of simpler cases done with S-PPV. This positions PFO use in E-PPV as a valuable tool during complex retinal detachment repair. Larger prospective studies are needed.

## Supplementary Information

Below is the link to the electronic supplementary material.

Supplementary Material 1

## Data Availability

Relevant data can be found within the manuscript tables, but additional datasets are available from the corresponding author on reasonable request.

## Abbreviations

RD: Retinal detachment
PFCL: Perfluorocarbon liquid
PFO: Perfluoro-n-octane
PPV: Pars plana vitrectomy
S-PPV: Surgical-microscope pars plana vitrectomy
E-PPV: Endoscopic pars plana vitrectomy
VA: Visual acuity
IOP: Intraocular pressure
CME: Cystoid macular edema
ERM: Epiretinal membrane

## References

[1] Mitry D, Charteris DG, Fleck BW, Campbell H, Singh J. The epidemiology of rhegmatogenous retinal detachment: geographical variation and clinical associations. Br J Ophthalmol. 2010;94(6):678–84. 19515646 10.1136/bjo.2009.157727

[2] D’Amico DJ. Clinical practice. Primary retinal detachment. N Engl J Med. 2008;359(22):2346–54. 19038880 10.1056/NEJMcp0804591

[3] Chang S. Perfluorocarbon liquids in vitreoretinal surgery. Int Ophthalmol Clin. 1992;32(2):153–63. 1577557 10.1097/00004397-199203220-00014

[4] Neffendorf JE, Gupta B, Williamson TH, The role of intraocular, gas tamponade in rhegmatogenous retinal detachment. A synthesis of the literature. Retina. 2018;38(Suppl 1):S65–72. 29280936 10.1097/IAE.0000000000002015

[5] Pastor JC, Coco RM, Fernandez-Bueno I, Alonso-Alonso ML, Medina J, Sanz-Arranz A, et al. Acute retinal damage after using a toxic perfluoro-octane for vitreo-retinal surgery. Retina. 2017;37(6):1140–51. 28538613 10.1097/IAE.0000000000001680

[6] Haliyur R, Portney DS, Pan WW, Mian SI, Rao RC. Cost drivers of pars plana vitrectomy for retinal detachment: time-driven activity-based costing analysis. J Vitreoretin Dis. 2024:24741264241279624. 10.1177/24741264241279624 PMC11556315 39539821

[7] Rodríguez-González F, Tejera-Santana M. Vitreous inflammation and macular edema secondary to perfluoro-n-octane toxicity. Rom J Ophthalmol. 2021;65(2):180–2. 34250315 10.22336/rjo.2021.35 PMC8207855

[8] Ajlan RS, Desai AA, Mainster MA. Endoscopic vitreoretinal surgery: principles, applications and new directions. Int J Retina Vitreous. 2019;5:15. 31236288 10.1186/s40942-019-0165-z PMC6580629

[9] Holladay JT. Proper method for calculating average visual acuity. J Refract Surg. 1997;13(4):388–91. 9268940 10.3928/1081-597X-19970701-16

[10] Tiew S, Lim C, Sivagnanasithiyar T. Using an excel spreadsheet to convert Snellen visual acuity to LogMAR visual acuity. Eye. 2020;34(11):2148–9. 32020059 10.1038/s41433-020-0783-6 PMC7784686

[11] Grover S, Fishman GA, Anderson RJ, Tozatti MS, Heckenlively JR, Weleber RG, et al. Visual acuity impairment in patients with retinitis pigmentosa at age 45 years or older. Ophthalmology. 1999;106(9):1780–5. 10485550 10.1016/S0161-6420(99)90342-1

[12] Brant A, Kolomeyer N, Goldberg JL, Haller J, Lee CS, Lee AY, et al. Evaluating visual acuity in the American academy of ophthalmology IRIS^®^ registry. Ophthalmol Sci. 2024;4(1):100352. 37869025 10.1016/j.xops.2023.100352 PMC10587626

[13] Elsing SH, Fekrat S, Green WR, Chang S, Wajer SD, Haller JA. Clinicopathologic findings in eyes with retained perfluoro-n-octane liquid. Ophthalmology. 2001;108(1):45–8. 11150263 10.1016/s0161-6420(00)00481-4

[14] Chang S, Sparrow JR, Iwamoto T, Gershbein A, Ross R, Ortiz R. Experimental studies of tolerance to intravitreal perfluoro-n-octane liquid. Retina. 1991;11(4):367–74. 1813951

[15] Toffoli D, Arbour JD, Harasymowycz P. Retained perfluoron postvitreoretinal surgery causing secondary open-angle glaucoma. Can J Ophthalmol. 2008;43(3):372. 18443616 10.3129/i08-047

[16] Pradeep S, Chhablani JK, Patel B, Rani P. Delayed inflammation associated with retained perfluorocarbon liquid. Indian J Ophthalmol. 2011;59(5):396–8. 21836352 10.4103/0301-4738.83623 PMC3159328

[17] Scott IU, Flynn HW Jr., Murray TG, Feuer WJ. Outcomes of surgery for retinal detachment associated with proliferative vitreoretinopathy using perfluoro-n-octane: a multicenter study. Am J Ophthalmol. 2003;136(3):454–63. 12967798 10.1016/s0002-9394(03)00241-1

[18] Sigler EJ, Randolph JC, Calzada JI, Charles S. Pars plana vitrectomy with medium-term postoperative perfluoro-N-octane for recurrent inferior retinal detachment complicated by advanced proliferative vitreoretinopathy. Retina. 2013;33(4):791–7. 23117281 10.1097/IAE.0b013e31826a6978

