# Supplementary material for: Comparison of perfluorocarbon liquid utilization in endoscopic pars plana vitrectomy and microscopic pars plana vitrectomy during retinal detachment repair: a retrospective review
Source: Int J Retina Vitreous. 2026 Feb 12;12:35. doi: 10.1186/s40942-026-00802-9 (PMC12906078; doi:10.1186/s40942-026-00802-9)
Supplement: Supplementary file 1 — Supplementary Material 1 [file 40942_2026_802_MOESM1_ESM.docx]

**Supplementary Table 1.** LogMAR Visual Acuity for PFO and Non-PFO groups.

| Time from Repair | All LogMAR VA  (N = 401) | PFO LogMAR VA  (N = 117) | No PFO LogMAR VA  (N = 284) | *p*-value |
| --- | --- | --- | --- | --- |
| Pre-op | 1.44 | 1.71 | 1.31 | *p* < 0.001 |
| POD #1 | 2.13 | 2.15 | 2.12 | *p* = 0.729 |
| POW #1 | 1.71 | 1.98 | 1.59 | *p* < 0.001 |
| POM #1 | 1.20 | 1.42 | 1.08 | *p* = 0.001 |
| POY #1 | 1.02 | 1.27 | 0.91 | *p* = 0.023 |

POD = Postoperation day, POW = Postoperation week, POM = Postoperation month, POY = Postoperation year

**Supplementary Table 2.** LogMAR Visual Acuity for E-PPV with PFO and E-PPV without PFO groups.

| Time from Repair | E-PPV with PFO LogMAR BCVA  (N = 31) | E-PPV without PFO  LogMAR BCVA  (N = 49) | *p*-value |
| --- | --- | --- | --- |
| Pre-op | 2.23 | 1.73 | *p* = 0.025 |
| POD #1 | 2.30 | 2.12 | *p* = 0.162 |
| POW #1 | 2.14 | 1.80 | *p* = 0.074 |
| POM #1 | 1.96 | 1.26 | *p* = 0.003 |
| POY #1 | 1.84 | 1.23 | *p* = 0.065 |

POD = Postoperation day, POW = Postoperation week, POM = Postoperation month, POY = Postoperation year

**Supplementary Table 3.** LogMAR Visual Acuity for S-PPV with PFO and S-PPV without PFO groups.

| Time from Repair | S-PPV with PFO LogMAR BCVA  (N = 86) | S-PPV without PFO  LogMAR BCVA  (N = 235) | *p*-value |
| --- | --- | --- | --- |
| Pre-op | 1.50 | 1.19 | *p* = 0.026 |
| POD #1 | 2.09 | 2.12 | *p* = 0.971 |
| POW #1 | 1.92 | 1.54 | *p* = 0.219 |
| POM #1 | 1.24 | 1.04 | *p* = 0.429 |
| POY #1 | 1.04 | 0.83 | *p* = 0.889 |

POD = Postoperation day, POW = Postoperation week, POM = Postoperation month, POY = Postoperation year

**Supplementary Table 4.** LogMAR Visual Acuity for E-PPV with PFO and S-PPV without PFO groups.

| Time from Repair | E-PPV with PFO LogMAR BCVA  (N = 31) | S-PPV without PFO  LogMAR BCVA  (N = 235) | *p*-value |
| --- | --- | --- | --- |
| Pre-op | 2.23 | 1.19 | *p* <0.001 |
| POD #1 | 2.30 | 2.12 | *p* = 0.148 |
| POW #1 | 2.14 | 1.54 | *p* = 0.001 |
| POM #1 | 1.96 | 1.04 | *p* < 0.001 |
| POY #1 | 1.84 | 0.83 | *p* <0.001 |

POD = Postoperation day, POW = Postoperation week, POM = Postoperation month, POY = Postoperation year

**Supplementary Table 5.** Retinal Re-detachment Rate

|  | Re-detachment Rate, N (%) | | | | |
| --- | --- | --- | --- | --- | --- |
|  | Total | POD #1 | POW #1 | POM #1 | POY #1 |
| All (N = 401) | 32 (7.98) | 5 (1.25) | 4 (1.00) | 17 (4.24) | 6 (1.50) |
| PFO (N = 117) | 12 (10.26) | 2 (1.71) | 2 (1.71) | 6 (5.13) | 2 (1.71) |
| No PFO (N = 284) | 20 (7.04) | 3 (1.06) | 2 (0.70) | 11 (3.87) | 4 (1.41) |
| E-PPV with PFO  (N = 31) | 4 (12.90) | 0 (0.00) | 0 (0.00) | 2 (6.45) | 2 (6.45) |
| S-PPV with PFO  (N = 86) | 10 (11.63) | 2 (2.33) | 2 (2.33) | 4 (4.65) | 2 (2.33) |
| E-PPV without PFO  (N = 49) | 1 (2.04) | 1 (2.04) | 0 (0.00) | 0 (0.00) | 0 (0.00) |
| S-PPV without PFO  (N = 235) | 19 (8.09) | 2 (0.85) | 2 (0.85) | 11 (4.68) | 4 (1.70) |
